# Right Ventricular Function and Pulmonary Arterial Coupling in Paediatric Obstructive Sleep Apnea

**DOI:** 10.1111/echo.70590

**Published:** 2026-08-12

**Authors:** Yumi Yuk‐ting Chan, Samuel Chung‐sum Ho, Chun‐ting Au, Dennis Lip‐yen Lee, Natalie Moon‐wah Leung, Ka‐li Kwok, Jacob Pak‐lam Ho, Hei‐to Leung, Kwok‐lap Chan, Albert Martin Li, Kate Ching‐ching Chan, Yiu‐fai Cheung

**Affiliations:** ^1^ Department of Paediatrics and Adolescent Medicine School of Clinical Medicine LKS Faculty of Medicine The University of Hong Kong Hong Kong China; ^2^ Department of Paediatrics and Adolescent Medicine Hong Kong Children's Hospital Hong Kong China; ^3^ Department of Paediatrics The Chinese University of Hong Kong Hong Kong China; ^4^ Department of Otorhinolaryngology Head and Neck Surgery The Chinese University of Hong Kong Hong Kong China; ^5^ Department of Paediatrics Kwong Wah Hospital Hong Kong China

**Keywords:** adenotonsillectomy, obstructive sleep apnea, right ventricle, right ventricular‐pulmonary arterial coupling

## Abstract

**Purpose:**

Right ventricular (RV) dysfunction and increased pulmonary arterial (PA) pressure may occur in children with obstructive sleep apnea (OSA). Long‐term data on RV‐PA coupling in children with OSA with or without adenotonsillectomy are absent.

**Methods:**

This prospective cohort study included children aged 5 to 12 years diagnosed with tonsillar hypertrophy and moderate‐to‐severe OSA. All subjects underwent echocardiographic assessment and sleep study at least 5 years since initial recruitment. Right atrial, RV function and indexed pulmonary artery acceleration time (PAATi) were evaluated. RV‐PA coupling was assessed by tricuspid annular plane systolic excursion (TAPSE) / PAATi and RV global longitudinal strain (GLS) / PAATi.

**Results:**

We evaluated 160 subjects, including 38 with OSA who declined adenotonsillectomy (Group I), 80 with OSA post‐adenotonsillectomy (Group II), and 42 controls (Group III). Baseline demographics were similar across groups. At a mean follow‐up of 7.2 ± 2.5 years, the latest OAHI did not differ significantly between the two OSA groups. Echocardiographic assessment revealed small intergroup differences in RA total strain, peak tricuspid annular systolic velocity, RVGLS and PAATi, whereas TAPSE, RV fractional area change and systolic strain rate were comparable across groups. Importantly, RV‐PA coupling indices did not differ significantly among the three groups. Furthermore, within the OSA cohort, neither follow‐up duration nor current OAHI severity correlated significantly with RV function or RV‐PA coupling indices.

**Conclusions:**

RV function and RV‐PA coupling are preserved in adolescents and young adults with a childhood diagnosis of OSA, irrespective of prior adenotonsillectomy.

## Graphical abstract

This prospective cohort study included 38 children with obstructive sleep apnea (OSA) who declined adenotonsillectomy, 80 with OSA post‐adenotonsillectomy, and 42 controls. At a mean follow‐up of 7.2 ± 2.5 years, echocardiographic assessment demonstrated preserved right ventricular systolic function and right ventricular‐pulmonary arterial coupling in adolescents and young adults with a childhood diagnosis of OSA, irrespective of prior adenotonsillectomy.

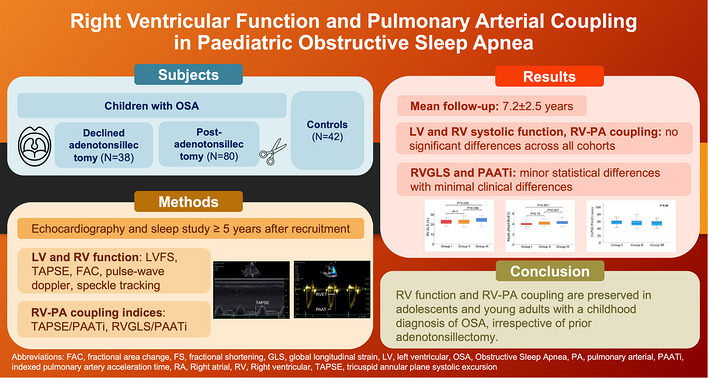

**Abbreviations**
A: Late Diastolic Tricuspid Inflow Velocity
a: tricuspid annular late diastolic velocity
AT: Adenotonsillectomy
BMI: Body Mass Index
BP: Blood Pressure
DBP: Diastolic Blood Pressure
E: Early Diastolic Tricuspid Inflow Velocity
e: tricuspid annular early diastolic velocity
EDA: End‐diastolic Area
EDD: End‐diastolic Dimension
ESA: End‐systolic Area
ESD: End‐systolic Dimension
FAC: Fractional Area Change
FS: Fractional Shortening
GLS: Global Longitudinal Strain
LV: Left Ventricular
mPAP: mean pulmonary artery pressure
MPI: Myocardial Performance Index
OAHI: Obstructive Apnea‐hypopnoea Index
OSA: Obstructive Sleep Apnea
PAAT: Pulmonary Artery Acceleration Time
PAATi: Indexed Pulmonary Artery Acceleration Time
RA: Right Atrial
RV: Right Ventricular
RVET: Right Ventricular Ejection Time
RV‐PA: Right Ventricular‐pulmonary Artery
s: peak tricuspid annular systolic velocity
SBP: Systolic Blood Pressure
SRa: Late Diastolic Strain Rate
SRe: Early Diastolic Strain Rate
SRs: Systolic Strain Rate
TAPSE: Tricuspid Annular Plane Systolic Excursion

## 1 Introduction

Obstructive sleep apnea (OSA) is a common condition in childhood and is frequently attributed to adenotonsillar hypertrophy [1]. While the cardiovascular consequences of OSA in adults are well established [2], there is growing evidence of its adverse impact on the vascular and cardiac function in the paediatric population. In children, OSA has been associated with systemic hypertension, cardiac arrhythmias, left ventricular (LV) hypertrophy, and LV dysfunction [3]. There is also evidence, albeit limited, to suggest the presence of right ventricular (RV) dysfunction and elevated pulmonary arterial pressure in these children [3, 4].

In children with OSA attributable to adenotonsillar hypertrophy, adenotonsillectomy (AT) remains to be the first‐line treatment [1, 3, 5]. While AT has been reported to improve LV function and hypertension in children with OSA [3], studies on the impact of AT on RV function and pulmonary pressure in these patients have yielded inconsistent results [5, 6, 7].

Assessment of RV performance should be made in the context of the pulmonary arterial system that it supports. Right ventricular‐pulmonary artery (RV‐PA) coupling refers to the dynamic interaction between the two components of the right‐sided circulation and reflects the ability of the right ventricle to adapt its output to its afterload [8]. The clinical significance of RV‐PA coupling has increasingly been recognized in different cardiovascular conditions including pulmonary hypertension [9], repaired tetralogy of Fallot [10], and heart failure [11, 12]. Increased pulmonary arterial load has been documented in OSA [2, 3, 4]. Nonetheless, RV‐PA coupling and the long‐term impact of AT on this phenomenon have hitherto not been explored in paediatric OSA.

The present study aimed to compare the RV function and RV‐PA coupling indices in adolescents and young adults with OSA, both with and without prior AT. We hypothesized that OSA patients who had not undergone AT would exhibit poorer RV function and RV‐PA coupling compared to those who had undergone AT and healthy subjects.

## 2 Methods

### 2.1 Subjects

This prospective study included children aged 5 to 12 years diagnosed with tonsillar hypertrophy and moderate‐to‐severe OSA, defined as an obstructive apnea‐hypopnea index (OAHI) of ≥ 3 events per hour. A control group of healthy children, confirmed to be free of OSA via overnight polysomnography, was also included. All subjects were invited to attend echocardiographic assessment and sleep study at least 5 years since initial recruitment. Exclusion criteria included i) history of additional upper airway surgery other than the procedure performed, ii) presence of cardiovascular conditions, and iii) current use of cardiovascular medications. The following data were retrieved from the medical notes: age at diagnosis, duration from baseline to follow‐up visits, baseline OAHI, the most recent OAHI and current use of nasal corticosteroids.

All subjects underwent echocardiographic assessment and sleep study as described below. Their body weight and height were measured, and the body mass index and body surface area were calculated accordingly. The blood pressure (BP) was measured in the right upper arm three times using an automated oscillometric device and the average was used for analysis. The study was approved by the Institutional Review Board, and adult subjects and parents of minors gave written informed consent.

### 2.2 Echocardiographic Assessment

All echocardiographic assessments were performed using Vivid E95 ultrasound machine (GE Medical System, Horten, Norway). The echocardiographic data were stored on digital versatile discs for offline analysis using EchoPAC software version 201. The average of echocardiographic indices measured from three cardiac cycles was used for subsequent analyses.

From the apical four‐chamber view, the RV end‐diastolic (EDA) and end‐systolic (ESA) areas were measured by two‐dimensional planimetry. RV fractional area change (FAC) was calculated as (EDA‐ESA)/EDAx100%. Tricuspid annular plane systolic excursion (TAPSE) was measured using M‐mode echocardiography and defined as the systolic displacement of the lateral tricuspid annulus toward the apex, a parameter with high feasibility and reproducibility in paediatric subjects [13]. LV end‐systolic and end‐diastolic dimensions were also measured, and LV fractional shortening (FS) was calculated. The severity of tricuspid regurgitation was assessed semi‐quantitatively using colour Doppler flow mapping.

Pulsed‐wave Doppler imaging was used for determination of trans‐tricuspid early (E), late (A) diastolic velocities and E/A ratio. Pulmonary artery acceleration time (PAAT), defined as the interval from the onset of pulmonary arterial systolic flow to peak velocity, and RV ejection time (RVET), defined as the interval from the onset to the end of pulmonary arterial systolic flow, were measured [14]. Indexed PAAT (PAATi) was calculated as PAAT/RVET to account for heart rate variability. Tissue Doppler imaging was performed at the basal RV free wall‐tricuspid annular junction to assess the tricuspid annular systolic (s), early diastolic (e), and late diastolic (a) myocardial velocities.

Two‐dimensional speckle tracking echocardiography was used for assessment of right atrial (RA) and RV myocardial deformation as reported previously by our group [15]. Briefly, the P wave was used as the reference point for determination of peak RA positive and negative strain and total strain. For determination of RV deformation, the QRS complex was used as the reference point for measurement of RV global longitudinal strain (GLS), systolic (SRs), early diastolic (SRe) and late diastolic (SRa) strain rates.

### 2.3 Assessment of RV‐PA Coupling

Right ventricular‐pulmonary arterial (RV‐PA) coupling was assessed using the TAPSE/PAATi and RVGLS/PAATi indices [8, 16, 17], which integrate surrogates of RV contractility (TAPSE and RVGLS) with a surrogate of RV afterload (PAATi). Although the TAPSE/systolic pulmonary arterial pressure (sPAP) ratio is widely used as a RV‐PA coupling index in previous studies [8, 18], data on its correlation with invasive catheterization indices in paediatric subjects are limited [16]. A possible reason is that this ratio depends on accurate estimation of sPAP via the modified Bernoulli equation based on the complete Doppler envelop of the tricuspid regurgitation jet, which is frequently absent or incomplete in patients and controls. Therefore, instead of using the TAPSE/sPAP index, we utilized PAAT that is a highly feasible and reproducible alternative [16]. PAAT is a validated estimate of RV afterload [8, 16], and the TAPSE/PAAT ratio has been documented as a reliable measure of RV‐PA coupling in both adult and paediatric populations [16]. We further utilized RV GLS, a sensitive marker of global RV function, as an additional surrogate of RV contractility for the assessment of RV‐PA coupling.

### 2.4 Statistical Analysis

Results are presented as mean±SD and median and range where appropriate. Absolute values of strain and strain rates are used for statistical analyses. The study cohort was categorized into three groups based on the OSA and AT status. Group I included subjects with OSA who declined AT, group II comprised subjects with OSA who had undergone AT, and group III consisted of control subjects. The demographic data and echocardiographic parameters were compared among three groups using one‐way ANOVA, with post‐hoc pairwise comparisons by Tukey test. Baseline and latest OAHI were compared between group I and II using Mann‐Whitney U test. Categorical variables were compared using Fisher's exact test. Subgroup analysis of group I and II subjects, based on the latest OAHI, with comparison of their echocardiographic parameters was performed using unpaired *t*‐test. Associations between demographic and clinical variables with indices of RV systolic function and RV‐PA coupling were assessed using Pearson correlation analysis for normally distributed continuous variables and Spearman rank correlation for non‐normally distributed variables. Multiple linear regression analysis was performed to identify significant correlates of RV systolic and RV‐PA coupling indices. A *p* value <0.05 was considered statistically significant. All statistical analyses were conducted using IBM SPSS Statistics 26 (SPSS, Chicago, Illinois, USA).

## 3 Results

### 3.1 Subjects

A total of 160 subjects were included in the study. Group I consisted of 38 subjects with OSA who declined AT, Group II comprised 80 subjects with OSA who had undergone AT, and Group III included 42 control subjects. Table 1 summarizes the demographic data, blood pressure and clinical characteristics of the three groups. There were no significant differences among the groups in terms of age, sex, body weight, height, body mass index (BMI), and BP (all *p* >0.05). Additionally, no significant differences in age at diagnosis, follow‐up duration, or baseline OAHI were observed between Groups I and II (all *p* >0.05). Overnight polysomnography was repeated in 97% (37/38) of Group I and 88% (70/80) of Group II subjects at a mean of 7.2 ± 2.5 years following their initial polysomnographic study. No significant differences in the latest OAHI were observed between the two groups (*p* = 0.81).

**TABLE 1:** Demographic parameters and clinical data.

	Group I (*N* = 38)	Group II (*N* = 80)	Group III (*N* = 42)	*P* (ANOVA)
**Demographic parameters**				
Age (years)	16.2 ± 4.0	16.2 ± 2.4	17.2 ± 2.9	0.19
Sex (M:F)	27:11	58:22	27:15	0.63
Weight (kg)	60.1 ± 15.6	61.9 ± 14.7	59.8 ± 16.7	0.73
Height (cm)	164.4 ± 9.4	167.5 ± 8.8	164.7 ± 9.9	0.13
BMI (kg/m^2^)	22.0 ± 4.4	21.9 ± 4.1	21.9 ± 5.1	0.99
**Blood pressure**				
SBP (mmHg)	114 ± 10	115 ± 15	114 ± 14	0.74
DBP (mmHg)	63 ± 6	64 ± 7	64 ± 6	0.78
**Clinical data**				
Age at diagnosis (years)	8.7 ± 1.8	8.6 ± 1.5		0.81
Duration from baseline to follow‐up visits (years)	7.5 ± 3.1	7.6 ± 2.0		0.80
Duration from adenotonsillectomy to follow‐up visits (years)		7.1 ± 2.0		
Baseline OAHI (events/h)	8.2 (3.0–60.0)	8.0 (3.0–78.0)		0.22
Severity group				0.09
<5 events/h	13 (34.2%)	14 (19.4%)		
≥5 events/h	25 (65.8%)	58 (80.6%)		
Latest OAHI (events/h)	3.5 (0.1–105.5)	3.2 (0.1–43.4)		0.81
Severity group				0.48
<5 events/h	24 (64.9%)	50 (71.4%)		
≥5 events/h	13 (35.1%)	20 (28.6%)		
Current use of nasal corticosteroids	5 (13.2%)	14 (17.5%)		0.54

Abbreviation: BMI, body mass index; DBP, diastolic blood pressure; OAHI, obstructive apnea‐hypopnea index; SBP, systolic blood pressure.

### 3.2 Echocardiographic Findings

Table 2 presents the echocardiographic findings across the three subject groups.

**TABLE 2:** Echocardiographic parameters.

	Group I (*N* = 38)	Group II (*N* = 80)	Group III (*N* = 42)	*P* (ANOVA)	I vs III	II vs III	I vs II
**Left ventricular indices**							
*M‐mode*							
LV EDD (mm/m^2^)	48.0 ± 4.1	48.0 ± 3.6	46.1 ± 4.0	0.021^*^	0.07	0.026^*^	1.00
LV ESD (mm/m^2^)	30.7 ± 3.7	30.9 ± 3.2	29.1 ± 3.5	0.021^*^	0.13	0.021^*^	1.00
FS (%)	36.2 ± 4.6	35.8 ± 4.6	36.8 ± 4.3	0.47			
**Right ventricular indices**							
***M‐mode***							
TAPSE (mm)	22.7 ± 2.9	22.7 ± 2.9	22.8 ± 2.6	0.99			
*2D echocardiography*							
RV ESA (cm^2^)	9.9 ± 1.8	10.6 ± 1.9	10.3 ± 2.0	0.24			
RV EDA (cm^2^)	16.8 ± 3.2	18.0 ± 3.4	17.7 ± 3.4	0.18			
RV FAC (%)	40.9 ± 2.9	41.3 ± 3.0	41.6 ± 2.7	0.57			
*Colour flow doppler*							
Tricuspid regurgitation (trivial/mild)	2 (5.3%)	10 (12.5%)	4 (9.5%)	0.47			
***Pulsed Doppler indices***							
E (cm/s)	56.5 ± 11.6	58.7 ± 10.5	59.6 ± 10.2	0.40			
A (cm/s)	37.8 ± 11.7	34.8 ± 9.1	35.6 ± 9.3	0.31			
E/A ratio	1.6 ± 0.6	1.8 ± 0.5	1.8 ± 0.5	0.40			
PAAT (ms)	122.2 ± 11.4	129.9 ± 11.2	138.5 ± 13.8	< 0.001^*^	< 0.001^*^	< 0.001^*^	0.004^*^
RVET (ms)	308.9 ± 23.2	317.0 ± 28.3	322.9 ± 24.9	0.06			
PAATi	0.40 ± 0.03	0.41 ± 0.03	0.43 ± 0.04	< 0.001^*^	< 0.001^*^	0.027^*^	0.15
***Tissue Doppler imaging***							
s (cm/s)	9.7 ± 1.5	10.4 ± 1.5	10.5 ± 1.4	0.021^*^	0.032^*^	1.00	0.047^*^
e (cm/s)	11.0 ± 1.9	11.8 ± 2.3	12.5 ± 2.0	0.007^*^	0.005^*^	0.27	0.14
a (cm/s)	8.0 ± 2.6	7.7 ± 2.7	7.8 ± 2.3	0.88			
**Speckle tracking**							
***RV deformation***							
GLS (%)	22.7 ± 2.4	22.9 ± 2.7	24.2 ± 2.7	0.015^*^	0.028^*^	0.036^*^	1.00
SRs (/s)	1.2 ± 0.2	1.3 ± 0.2	1.3 ± 0.2	0.10			
SRe (/s)	1.7 ± 0.4	1.7 ± 0.4	1.9 ± 0.4	0.010^*^	0.046^*^	0.011^*^	1.00
SRa (/s)	0.9 ± 0.3	0.8 ± 0.2	0.9 ± 0.4	0.41			
***RA deformation***							
Positive (%)	28.6 ± 2.9	28.6 ± 2.7	29.1 ± 2.5	0.60			
Negative (%)	14.3 ± 2.8	15.0 ± 2.2	15.6 ± 2.1	0.07			
Total (%)	42.9 ± 2.9	43.6 ± 3.0	44.7 ± 3.1	0.033^*^	0.030^*^	0.23	0.66
**RV‐PA coupling**							
TAPSE/PAATi (mm)	57.4 ± 7.3	55.6 ± 8.6	53.4 ± 7.6	0.08			
RVGLS/PAATi (%)	57.5 ± 8.0	56.2 ± 7.5	56.6 ± 7.2	0.70			

Abbreviations: A, late diastolic tricuspid inflow velocity, a, tricuspid annular late diastolic velocity, E, early diastolic tricuspid inflow velocity, e, tricuspid annular early diastolic velocity, EDA, end‐diastolic area, EDD, end‐diastolic dimension, ESA, end‐systolic area, ESD, end‐systolic dimension, FAC, fractional area change, FS, fractional shortening, GLS, global longitudinal strain, LV, left ventricular, PA, pulmonary arterial, PAAT, pulmonary artery acceleration time, PAATi, indexed pulmonary artery acceleration time, RA, right atrial, RV, right ventricular, RVET, right ventricular ejection time, s, peak tricuspid annular systolic velocity, SRa, late diastolic strain rate, SRe, early diastolic strain rate, SRs, systolic strain rate, TAPSE, tricuspid annular plane systolic excursion.

^*^Statistically Significant

Regarding LV systolic function, no significant differences in LVFS were observed among the groups (*p* = 0.47).

**FIGURE 1:**
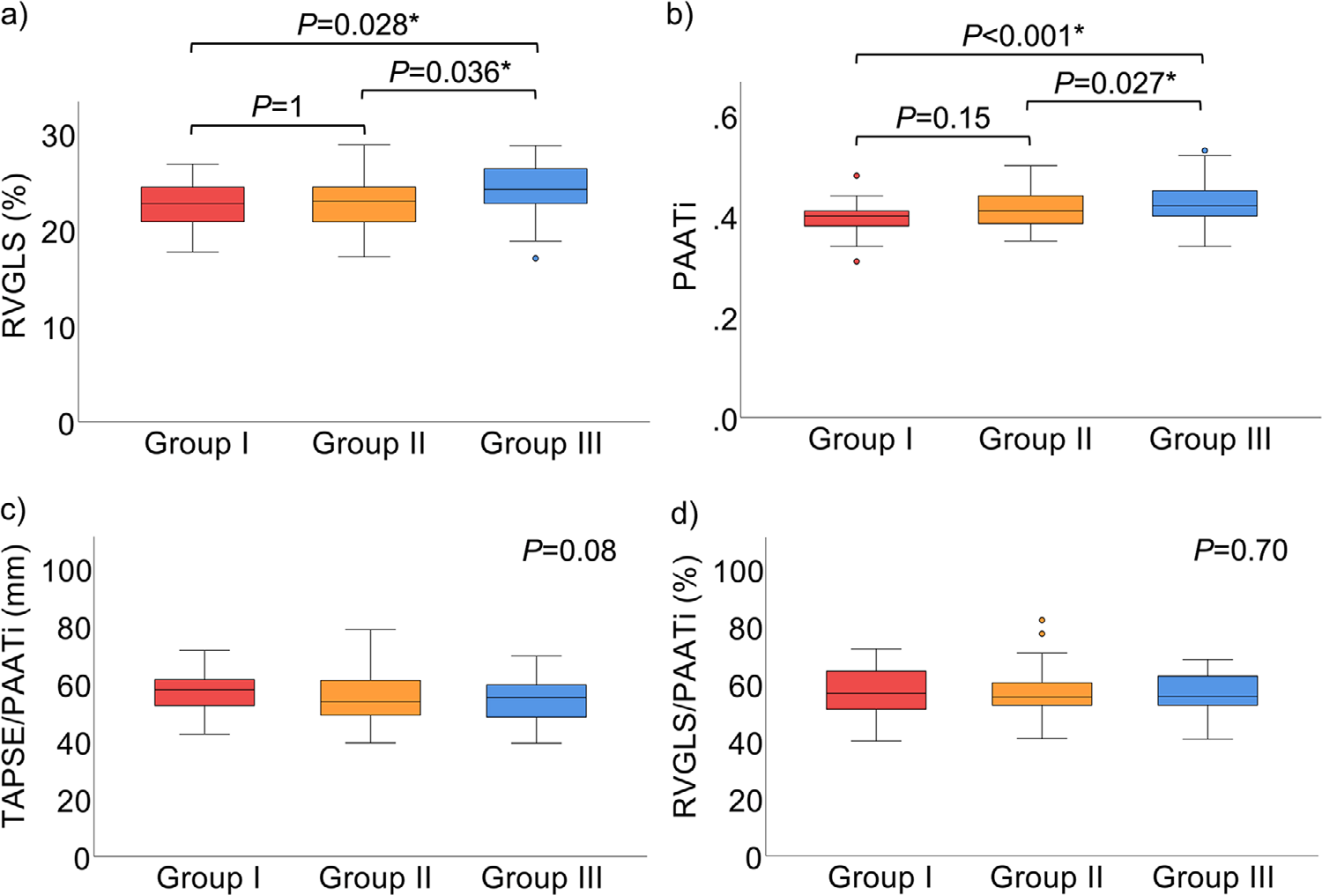
Boxer and whisker plots showing (a) RVGLS, (b) PAATi, (c) TAPSE/PAATi and (d) RVGLS/PAATi in the three groups. GLS, global longitudinal strain, PAATi, indexed pulmonary artery acceleration time adjusted for heart rate, RV, right ventricular, TAPSE, tricuspid annular plane systolic excursion.

For RV systolic functional indices, TAPSE, RVFAC and SRs showed no significant differences among the three groups (all *p* >0.05). Although the difference in tricuspid s velocity was statistically significant (*p* = 0.021), it was clinically small in magnitude. Similarly, for RVGLS, minor but statistically significant differences were noted between Group I and III (*p* = 0.028) and between Group II and III (*p* = 0.036), and no significant difference was found between Group I and II (*p* = 1.00; Figure 1a). For RV diastolic parameters, there was no significant difference in SRa (*p* = 0.41), whereas a small but statistically significant difference was observed in SRe (*p* = 0.010).

Regarding RA speckle tracking parameters, no significant differences were noted in RA peak positive (*p* = 0.60) and negative (*p* = 0.07) strains. A small but significant difference was observed in RA total strain across groups (*p* = 0.033).

Trivial or mild tricuspid regurgitation was present in 5.3% (2/38) of Group I, 12.5% (10/80) of Group II, and 9.5% (4/42) of Group III, with no significant differences among the groups (*p* = 0.47). For PAAT, significant differences were observed across groups (*p* < 0.001). However, after correction for heart rate variability, the absolute differences in PAATi among the three groups were minimal, despite remaining statistically significant (*p* < 0.001; Figure 1b). Post‐hoc comparison demonstrated significant differences between Group I and III (*p* < 0.001) and between Group II and III (*p* = 0.027) with no significant difference between Group I and II (*p* = 0.15).

### 3.3 RV‐PA Coupling

As illustrated in Figures 1c–d, no significant differences were observed across the three groups for either TAPSE/PAATi (*p* = 0.08) or RVGLS/PAATi (*p* = 0.70).

### 3.4 Subgroup Analysis

**FIGURE 2:**
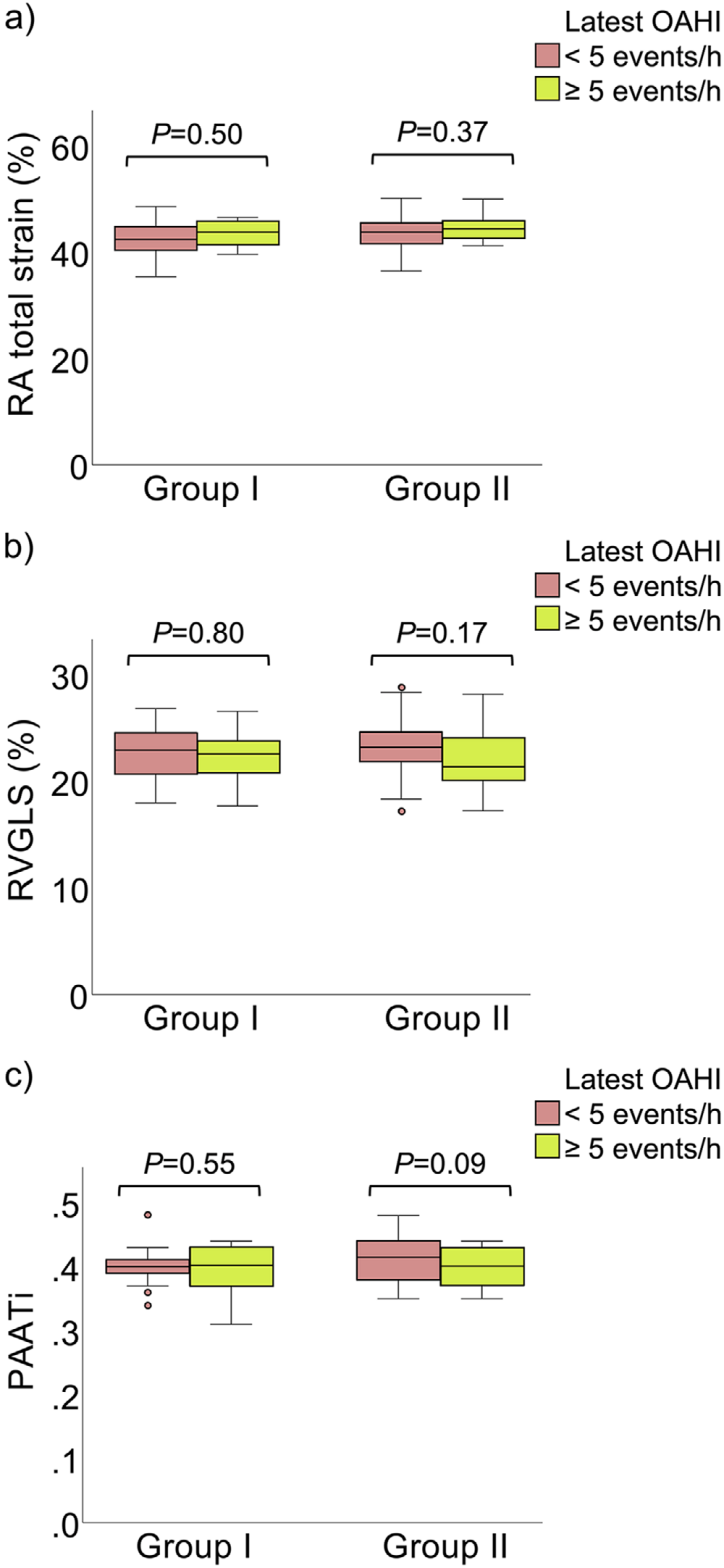
Subgroup analysis of group I and II patients stratified by the latest OAHI. The boxer and whisker plots showing (a) RA total strain, (b) RV GLS, and (c) PAATi in patients with OAHI< 5 events/hour and those with OAHI ≥ 5 events/hour. GLS, global longitudinal strain, OAHI, obstructive apnea‐hypopnea index, PAATi, indexed pulmonary artery acceleration time adjusted for heart rate, RA, right atrial, RV, right ventricular.

Subgroup analyses for Groups I and II were conducted by stratifying subjects based on the latest OAHI (≥5 events/hour versus < 5 events/hour). In both groups, no statistically significant differences in RA total strain, RVGLS and PAATi were found between the subgroups (all *p* >0.05, Figure 2a–c, Supplementary Table 1).

A further subgroup analysis combined subjects from Groups I and II, stratifying them using the same threshold for latest OAHI. This yielded 69 subjects with a latest OAHI of ≥5 events/hour and 26 subjects with a latest OAHI of < 5 events/hour. Similarly, no statistically significant differences were observed between the subgroups (all *p* >0.05, Supplementary Table 1).

### 3.5 Correlations of RV GLS and RV‐PA Coupling Indices

Univariate and multivariate analyses were performed to identify factors associated with RVGLS and RV‐PA coupling indices (Supplementary Table 2). Subject grouping was the only significant, albeit weak, correlate of RVGLS (β = 0.21, *p* = 0.010) and TAPSE/PAATi (β = −0.18, *p* = 0.027). Within the OSA patient cohort alone, no significant correlations were found between follow‐up duration or the latest OAHI with RVGLS and RV‐PA coupling indices (all *p* >0.05).

## 4 Discussion

The primary finding of this study is the preservation of RV function and RV‐PA coupling in adolescents and young adults with a childhood diagnosis of OSA, irrespective of prior AT. Although several echocardiographic parameters reached statistical significance, the reported differences are small and of uncertain clinical relevance. Furthermore, we found no evidence of elevated pulmonary pressures within our OSA cohort, and RV‐PA coupling remained comparable across all three study groups. To our knowledge, this is the first study to evaluate RV‐PA coupling and to assess the long‐term impact of AT on this coupling phenomenon in paediatric patients with OSA.

Previous studies evaluating RV function in children with OSA have yielded conflicting results. While some reported similar TAPSE between OSA patients and healthy controls [7], others observed lower TAPSE in the OSA cohort [19, 20]. The reliance on RV myocardial performance index (MPI) in previous studies [7, 19, 21], a parameter with limited reproducibility in paediatric populations [22], may have further contributed to the inconsistent results. Compared to the above previous studies, the present study features a larger sample size, a longer follow‐up duration and a predominantly adolescent cohort rather than young paediatric subjects. We found no significant differences in conventional RV functional parameters such as TAPSE and RVFAC. RVGLS was also included in our analysis, as it has demonstrated higher sensitivity than conventional RV functional parameters for detecting subclinical cardiac damage and early disease in adult patients with OSA [23]. However, in contrast to the adult data [23], we demonstrated only a minimal and clinically insignificant difference in RV GLS between paediatric OSA patients and healthy controls, suggesting that global RV function is largely preserved in this cohort, even when assessed using the more sensitive index of RV myocardial deformation. Additionally, regarding the impact of AT on RV function, previous paediatric studies have reported inconsistent outcomes, with some finding no difference in RV MPI [7, 8] while others noting higher TAPSE in patients who had undergone AT [19, 20]. In our cohort, however, we found no significant differences in any RV systolic or diastolic functional parameters between patients with and without prior AT. Potential explanations for the preserved RV function in our cohort are detailed below, specifically in the context of pulmonary pressure and RV‐PA coupling.

In this study, pulmonary arterial pressure was non‐invasively estimated using established echocardiographic parameters. In our cohort, tricuspid regurgitation jets were at most trivial to mild, rendering the estimation of sPAP challenging due to incomplete Doppler envelopes. Previous paediatric studies have also utilized the Mahan formula, an index that incorporates PAAT, to estimate mean pulmonary artery pressure (mPAP) in patients with OSA [5]. Using this method, Cetin et al. found significantly higher mPAP in children with OSA compared to healthy controls [19], while Cincin et al. noted improvement in mPAP after AT [7]. However, absolute PAAT requires careful interpretation in children due to its dependence on heart rate. After adjusting for heart rate, we observed only minimal differences in PAATi among the three study groups. Notably, all PAATi values remained within the normal paediatric limits (> 0.29) [14], indicating no evidence of pulmonary hypertension among our subjects.

To the best of our knowledge, this is the first study to evaluate RV‐PA coupling during long‐term follow‐up in children with OSA. RV‐PA coupling has traditionally been assessed invasively by right heart catheterization using the ratio of end‐systolic elastance (Ees) to arterial elastance (Ea) derived from the pressure‐volume loops [8]. In the present study, we assessed RV‐PA coupling non‐invasively using echocardiographic indices TAPSE/PAATi and RVGLS/PAATi. Levy et al. previously reported a strong correlation between TAPSE/PAAT and the invasively derived RV Ees/Ea ratio in a paediatric cohort with an interquartile age range of 1.3 to 12.6 years [16]. We used PAATi, rather than PAAT, as a surrogate for RV afterload to account for heart rate variability in children. We also included RVGLS as an additional surrogate of RV contractility because it is a more sensitive marker of global RV function than conventional indices such as TAPSE [23]. None of these RV‐PA coupling indices differed significantly among the three study groups, suggesting that the relationship between RV contractility and pulmonary arterial load remained preserved in children with OSA.

The preservation of both RV function and RV‐PA coupling in our cohort may possibly be explained by the absence of significant pulmonary hypertension. Although adenotonsillar hypertrophy causes intermittent airway obstruction and chronic alveolar hypoventilation, which can eventually lead to elevated pulmonary artery pressure and progressive RV dysfunction [5], these sequelae are not evident in our study patient cohorts. Consequently, clinically significant pulmonary hypertension in the setting of OSA is far more prevalent in adults than in children [24, 25]. The absence of pulmonary hypertension in our paediatric cohort has spared the right ventricle from any sustained increase in afterload, preserving both its function and its coupling mechanics at this early stage of disease. Additionally, we observed spontaneous improvement in OSA severity within our untreated cohort (Group I), a phenomenon reported previously and attributed to progressive tonsillar regression and airway enlargement as children grow [26]. This may further account for the comparable RV function and pulmonary pressures between our two OSA groups.

Key strengths of this study include its longitudinal prospective design, extended follow‐up period, a relatively large paediatric sample size, and the use of comprehensive echocardiographic assessments alongside overnight polysomnography at both baseline and follow‐up. The observed preservation of RV function and RV‐PA coupling in our cohort suggests that further invasive cardiovascular assessment is unwarranted at this early stage. However, as the risk of developing pulmonary hypertension and RV dysfunction may evolve with age, continued longitudinal monitoring into adulthood remains essential.

This study has several limitations. First, the study design limits our ability to determine the prognostic significance of our findings. Second, the absence of baseline echocardiographic data at the time of initial subject recruitment precludes assessment of the longitudinal effects of AT on cardiovascular parameters. Third, we relied on a non‐invasive assessment of RV‐PA coupling indices at a resting state. It remains possible that dynamic assessment during exercise stress could unmask subclinical pulmonary hypertension and reveal RV‐PA uncoupling, as has been demonstrated in patients with pulmonary arterial hypertension [27] and heart failure with preserved ejection fraction [28]. Fourth, patients who declined adenotonsillectomy may represent a selected population with different clinical characteristics, introducing potential selection bias. Nonetheless, as the decision to have adenotonsillectomy or not was made by parents rather than the attending clinicians, the influence of systematic selection bias on the observed findings is probably limited. Finally, the large number of statistical comparisons performed without adjustment for multiple testing increases the risk of type I error and may account for some findings of marginal statistical significance.

In conclusion, RV function and RV‐PA coupling are preserved in adolescents and young adults with a childhood diagnosis of OSA, irrespective of prior AT. Future longitudinal studies are warranted to elucidate the long‐term significance of these findings.

## Funding

This study was supported by the Health and Medical Research Fund provided by the Food and Health Bureau of the Hong Kong SAR, China (grant number: 07181276).

## Conflicts of Interest

All authors declare that they have no conflicts of interest.

## Institutional Review Board

This study was approved by Research Ethics Committees of the Hong Kong Children's Hospital (Reference number: HKCH‐REC‐2021‐011), Institutional Review Board of The University of Hong Kong/Hospital Authority Hong Kong West Cluster (Reference number: UW 19–718), The Joint CUHK‐NTEC Clinical Research Ethics Committee (Reference Number: 2019.218), Research Ethics Committees of Kowloon Central/Kowloon East (Reference Number: KC/KE‐19‐0250/ER‐3)

## Supporting information




**Supporting file 1**: echo70590‐sup‐0001‐FigureS1.docx

**Supporting file 1**: echo70590‐sup‐0002‐FigureS2.docx
