# Supplementary material for: Right Ventricular Function and Pulmonary Arterial Coupling in Paediatric Obstructive Sleep Apnea
Source: Echocardiography. 2026 Aug 12;43(8):e70590. doi: 10.1111/echo.70590 (PMC13465734; doi:10.1111/echo.70590)
Supplement: Supplementary file 1 [file ECHO-43-e70590-s001.docx]

**Supplementary table 1.** Subgroup analysis of group I and II patients stratified by the latest OAHI

|  | Group I |  |  |  | Group II |  |  |  | Group I and II | |  |
| --- | --- | --- | --- | --- | --- | --- | --- | --- | --- | --- | --- |
|  | Latest OAHI  ≥5 events/hr N=24 | Latest OAHI  <5 events/hr N=13 | *P* |  | Latest OAHI  ≥5 events/hr N=50 | Latest OAHI  <5 events/hr N=20 | *P* |  | Latest OAHI  ≥5 events/hr N=69 | Latest OAHI  <5 events/hr N=26 | *P* |
| RA total strain (%) | 42.6 ± 3.1 | 43.3 ± 2.6 | 0.50 |  | 43.6 ± 3.2 | 44.3 ± 2.4 | 0.37 |  | 43.3 ± 3.2 | 43.9 ± 2.5 | 0.33 |
| RVGLS (%) | 22.7 ± 2.5 | 22.4 ± 2.5 | 0.80 |  | 23.1 ± 2.6 | 22.0 ± 3.0 | 0.17 |  | 23.0 ± 2.5 | 22.2 ± 2.7 | 0.18 |
| PAATi | 0.40 ± 0.03 | 0.39 ± 0.04 | 0.55 |  | 0.41 ± 0.03 | 0.40 ± 0.03 | 0.09 |  | 0.41 ± 0.03 | 0.40 ± 0.03 | 0.08 |

Abbreviations: GLS, global longitudinal strain, OAHI, obstructive apnea-hypopnea index, PAATi, indexed pulmonary artery acceleration time, RA, right atrial, RV, right ventricular
