# Supplementary material for: Right Ventricular Function and Pulmonary Arterial Coupling in Paediatric Obstructive Sleep Apnea
Source: Echocardiography. 2026 Aug 12;43(8):e70590. doi: 10.1111/echo.70590 (PMC13465734; doi:10.1111/echo.70590)
Supplement: Supplementary file 2 [file ECHO-43-e70590-s002.docx]

**Supplementary Table 2**. Univariate and multivariate analyses of RVGLS and RV-PA coupling indices

|  | RVGLS | | | |  | TAPSE/PAATi | | | |  | RVGLS/PAATi | | | |
| --- | --- | --- | --- | --- | --- | --- | --- | --- | --- | --- | --- | --- | --- | --- |
|  | r | p | β | p |  | r | p | β | p |  | r | p | β | p |
| **Entire cohort** |  |  |  |  |  |  |  |  |  |  |  |  |  |  |
| Age | 0.06 | 0.45 | - | - |  | -0.01 | 0.90 | - | - |  | -0.09 | 0.28 | - | - |
| Sex | - | - | 0.04 | 0.60 |  | - | - | -0.01 | 0.92 |  |  |  | -0.01 | 0.94 |
| Group | - | - | 0.21 | 0.010* |  | - | - | -0.18 | 0.027* |  |  |  | -0.04 | 0.63 |
| BMI | -0.05 | 0.53 | - | - |  | 0.11 | 0.17 | - | - |  | -0.02 | 0.82 | - | - |
| SBP | 0.03 | 0.73 | - | - |  | 0.09 | 0.25 | - | - |  | 0.00 | 0.99 | - | - |
| DBP | -0.14 | 0.10 | - | - |  | -0.04 | 0.64 | - | - |  | -0.10 | 0.23 | - | - |
| **OSA patients** |  |  |  |  |  |  |  |  |  |  |  |  |  |  |
| Follow-up duration | -0.01 | 0.95 | - | - |  | 0.07 | 0.50 | - | - |  | -0.07 | 0.51 | - | - |
| Latest OAHI | -0.14 | 0.15 | - | - |  | -0.05 | 0.63 | - | - |  | -0.03 | 0.74 | - | - |

Abbreviations: BMI, body mass index, DBP, diastolic blood pressure, GLS, global longitudinal strain, OAHI, OAHI, obstructive apnea-hypopnea index, OSA, obstructive sleep apnea, PAATi, indexed pulmonary artery acceleration time, RV, right ventricular, SBP, systolic blood pressure, TAPSE, tricuspid annular plane systolic excursion

*Statistically significant
